# Identification of HDAC9 and ARRDC4 as potential biomarkers and targets for treatment of type 2 diabetes

**DOI:** 10.1038/s41598-024-57794-5

**Published:** 2024-03-25

**Authors:** Jing Liu, Lingzhen Meng, Zhihong Liu, Ming Lu, Ruiying Wang

**Affiliations:** 1 Endocrinology Department, The Second Hospital of Hebei Medical University, No.215 Heping West Road, Shijiazhuang, 050000 People’s Republic of China; 2 General Medical Department, The Fourth Hospital of Hebei Medical University, No.12 Jiankang Road, Shijiazhuang, 050000 People’s Republic of China; 3 Medical Department, The Second Hospital of Hebei Medical University, No.215 Heping West Road, Shijiazhuang, 050000 People’s Republic of China

**Keywords:** Type 2 diabetes, Insulin resistance, Immunotherapy, Diagnostic biomarker, Computational biology and bioinformatics, Genetics, Immunology, Biomarkers, Diseases, Endocrinology

## Abstract

We aimed to identify the key potential insulin resistance (IR)-related genes and investigate their correlation with immune cell infiltration in type 2 diabetes (T2D). The GSE78721 dataset (68 diabetic patients and 62 controls) was downloaded from the Gene Expression Omnibus database and utilized for single-sample gene set enrichment analysis. IR-related genes were obtained from the Comparative Toxicology Genetics Database, and the final IR-differentially expressed genes (DEGs) were screened by intersecting with the DEGs obtained from the GSE78721 datasets. Functional enrichment analysis was performed, and the networks of the target gene with microRNA, transcription factor, and drug were constructed. Hub genes were identified based on a protein–protein interaction network. Least absolute shrinkage and selection operator regression and Random Forest and Boruta analysis were combined to screen diagnostic biomarkers in T2D, which were validated using the GSE76894 (19 diabetic patients and 84 controls) and GSE9006 (12 diabetic patients and 24 controls) datasets. Quantitative real-time polymerase chain reaction was performed to validate the biomarker expression in IR mice and control mice. In addition, infiltration of immune cells in T2D and their correlation with the identified markers were computed using CIBERSORT. We identified differential immune gene set regulatory T-cells in the GSE78721 dataset, and T2D samples were assigned into three clusters based on immune infiltration. A total of 2094 IR-DEGs were primarily enriched in response to endoplasmic reticulum stress. Importantly, *HDAC9 and ARRDC4* were identified as markers of T2D and associated with different levels of immune cell infiltration. *HDAC9* mRNA level were higher in the IR mice than in control mice, while *ARRDC4* showed the opposite trend. In summary, we discovered potential vital biomarkers that contribute to immune cell infiltration associated with IR, which offers a new sight of immunotherapy for T2D.

## Introduction

Type 2 diabetes (T2D), namely non-insulin-dependent diabetes, is characterized by insulin resistance (IR) and incidence rates are increasing at an alarming speed^1^. Insulin resistance is an important pathological feature in the development of type 2 diabetes, which mainly refers to the inability of normal levels of insulin to induce the subsequent normal chain reaction after binding to insulin receptors on the surface of target tissues of the body (liver, fat and skeletal muscle). Many factors are involved in IR, such as inflammation^2^, oxidative stress^3^, and endoplasmic reticulum stress^4^, yet others remain largely unknown.

Inflammation is involved in the pathogenesis of insulin resistance. Increased secretion of inflammatory factors and massive infiltration of inflammatory cells contribute to the activation of inflammatory signaling pathways such as IKKβ/NF-κB and JNK, ultimately leading to insulin resistance and type 2 diabetes mellitus. Moreover, increasingly, studies are reporting on immune cells mediating inflammation-induced IR in T2D. A recent study indicates that increased levels of galectin-3 secreted from macrophages in obese humans and mice contribute to the inhibition of insulin signaling^2^. Consequently, regulating immune cell dysfunction may be an effective strategy to remit hepatic IR for T2D therapy^5^. However, the exact molecular mechanism regarding the activation of immune system-induced hepatic IR remains poorly understood.

Diabetes, as a chronic disease, may not have obvious early symptoms, but once diagnosed, it may have progressed to a more serious stage. The discovery and application of biomarkers can help provide effective interventions in the early stages of disease, thereby improving treatment outcomes and quality of life. By gaining a deeper understanding of these markers, we hope to not only better understand the pathogenesis of diabetes, but also be able to provide more targeted treatment options for individual patients. A better understanding of the immune mechanisms behind insulin resistance is critical for improving clinical outcomes in patients with T2D. Notably, there is limited information on the transcriptional profiles of T2D patients, which hampers the study of T2D pathogenesis and potential biomarkers. Therefore, identifying and characterising these potential T2D markers may improve clinical recovery rates. Recently, bioinformatics analyses have been important tools for diabetic research^6,7^. However, multi-platform analyses, different statistical methods, and a lack of important variables (e.g., different courses of disease) in the raw data have resulted in discordant findings. Further, few studies have used bioinformatics analysis to investigate the potential molecular mechanisms underlying immune infiltration characteristics in hepatic IR for T2D^8,9^. Accordingly, we have made a further comprehensive analysis.

Epigenetics is playing an increasingly important role in the study of type 2 diabetes, particularly in relation to HDAC (histone deacetylase). HDAC9, as a member of this group, is involved in the regulation of cellular gene expression, which is closely linked to the development of diabetes mellitus.

This investigation aimed to meet a pressing need to identify type 2 diabetes specific biomarkers. The prevalence of diabetes is increasing globally, there are substantial gaps in our understanding of the molecular mechanisms underlying insulin resistance and immune cell infiltration. We focussed on the analysis of gene expression data from microarrays of type 2 diabetes to elucidate genetic factors contribute to disease pathogenesis. The motivation to conduct this study originated from revealing potential impact of novel prognostic biomarkers, motivating our development of diagnostic and treatment strategies of type 2 diabetes. We believe that our study will offer new insights on the mechanisms of T2D for the development of therapeutic interventions.

## Materials and methods

### Data preprocessing

We employed the “GEOquery” package^10^ to retrieve three T2D microarray datasets —GSE78721^11^, GSE76894^12^, and GSE9006^13^—from the Gene Expression Omnibus (GEO) (https://www.ncbi.nlm.nih.gov/geo/) database. GEOquery is a toolkit for downloading and analysing gene expression data from the NCBI GEO database in R. It facilitates biologists and bioinformaticians to access and process gene expression data. Three GEO datasets were selected according to the species of the dataset, the number of samples, the chip platform, the source of the organization, and the quality of the dataset. Each dataset was grouped into either the T2D or normal group (Supplementary Table 1). The batch effects of the GSE78721 dataset were removed using the “sva” package^14^ (Supplementary Fig. 1). The sva package is an R package for detecting and adjusting for potential batch effects and other technical variations in high-dimensional gene expression data to improve the accuracy and reliability of expression data.

### Immune cell infiltration analysis based on the immune-related gene sets

Twenty-nine immune-related gene sets representing tumor immunity were identified via a published literature search^15^. We employed the gene set variation analysis “GSVA” package^16^ to obtain single-sample gene set enrichment analysis (ssGSEA) scores for the 29 immune-related gene sets associated with infiltration in the T2D samples in GSE78721. Differential expression analyses of ssGSEA enrichment scores were performed using the “limma” package^17^. Additionally, we used the “GOSemSim”^18^ package to compute their semantic similarity in biological processes.

### Identification of T2D subtypes based on immune cell gene sets

Consensus clustering and molecular subtype screening of the ssGSEA score were performed using ConsensusClusterPlus^19^. Consistent clustering, which is used to determine the optimal number of K-means clusters, is an algorithm that can be used to identify cluster members and their numbers in datasets such as microarray gene expression. Consistent clustering verifies the rationality of clustering by a resampling approach with the main purpose is to evaluate the stability of clustering. Consistent clustering methods involve sub-sampling from a set of items, such as a microarray, and identifying clusters with a specific cluster number (K). Then, for the consensus values, two items accounting for the same cluster in the number of occurrences in the same subsample are calculated and stored in a symmetric congruent matrix for each K. K is the specific cluster number. The optimal number of clusters was determined using the area under cumulative distribution function (CDF) curve. Two-dimensional principal component analysis (PCA) plots were used to assess the reliability of the consensus cluster.

### Identification of differentially expressed genes (DEGs)

Based on the GSE78721 grouping information, we applied the “limma” package to screen the difference between T2D samples and normal samples. *P*-value < 0.05 was set to identify significant DEGs. IR-DEGs were selected based on the intersection of DEGs and hepatic IR-related genes. Hepatic IR-related genes were gathered from the Comparative Toxicology Genetics Database (CTD).

### Functional enrichment analysis

The “clusterProfiler” package^20^ was used for Gene Ontology (GO) and Kyoto Encyclopedia of Genes and Genomes (KEGG)analysis. clusterProfiler is an R package for functional enrichment analysis and visualisation of gene sets in biological data to help researchers understand the biological meaning of gene sets. *P* < 0.05 was considered statistically significant. To investigate the biological processes enrichment in each sample, GSEA of GSE78721 was conducted using the “clusterProfiler” package^20^. Meanwhile, GSVA^16^ was performed using the “GSVA” package with 50 Hallmark gene sets from MSigDB^21^.

### Bioinformatics based Identification and validation of biomarkers.

A total of 130 samples in the GSE78721 dataset were further divided into training (*n* = 97) and test sets (*n* = 33) at a 7:3 ratio. First, we applied least absolute shrinkage and selection operator (LASSO) regression and Random Forest and Boruta (RFB) analysis to select the features for screening T2D biomarkers in the training sets. We then computed the significance scores of each gene via the “glmnet”^22^, “randomForest”^23^, and “Boruta” packages^24^ to acquire relevant characteristics. Next, we selected the expression of IR-DEGs as an input (independent variable), and disease state of the sample as an output (binary dependent variable, 0 or 1). In the training set, we assessed the appropriateness of the combined LASSO and RFB analysis for selecting characteristics using the receiver operating characteristic (ROC) curve and then computed the area under the curve (AUC). Finally, the “Re1071” package was used to construct a support vector machine (SVM) model possessing a radial basis function (RBF) kernel to verify the optimal feature genes^25^. We further validated the performance of the machine learning model using GSE76894 and GSE9006 as verification sets.

Twenty male 6-week-old C57BL/6 J mice, weighing around 22 g each, were purchased from Viton Lihua Experimental Center (Beijing, China) maintained on a standard 12 h/12 h light/dark cycle. The mice were equally divided into two groups: (*n* = 10 each), the control group and the IR group. The control mice were fed a routine diet, while the IR mice were fed a 60% high-fat diet (HFD) for 8 weeks to induce IR. The IR model was validated by determining the area under the curve in accordance with the protocol presented in our previously published study^26^. Insulin resistance was estimated using the quantitative insulin sensitivity check index (QUICKI).The mice were anesthetized, and their hepatic tissues were removed. Then, total RNA was extracted from mouse hepatic tissues using Trizol reagent (TIANGEN, Beijing, China). The RNA was reverse transcribed to cDNA according to the manufacturer's protocol and amplified by real-time PCR using the following primers: *HDAC9*, forward 5′- CAAAGATAGAGGACGAGAAAGGG-′3, reverse 5′- AGTTGGGCTCTGAGGCAGTTTT-′3; *ARRDC4*, forward 5′- CACGAGTTTCCCTTTCGCTTTC-′3, reverse 5′- ATAGGAGTCAATAAGGGCGGTGT -′3; β-actin, forward 5′- GGCGCTTTTGACTCAGGATT-′3, reverse 5′- GGGATGTTTGCTCCAACCAA -′3. Relative mRNA expression was calculated by the 2^-(ΔΔCt)^ method.

### Protein–protein interaction (PPI) network construction and hub gene identification

A PPI network was constructed using the STRING database (the confidence score cutoff was 0.4) and further visualized using “Cytoscape”^27^. Cytoscape is an open source software for biological network analysis and visualisation that integrates a wide range of bioinformatics data and graphically displays molecular interaction networks and biological pathways to facilitate systems biology research. The degree of each protein node in maximal clique centrality (MCC) was computed using cytoHubba and the top 20 MCC genes were selected as hub genes. Biological function enrichment analyses of the 20 hub genes were further performed using ClueGO^28^ and CluePedia^29^.

### Correlation analysis of immune infiltration and immune related genes

The expression of 20 hub genes was obtained from the expression profile. This information was used to further analyze the relationship of hub gene expression in immune signatures subtypes as well as the correlation between the 20 hub genes and the ssGSEA scores for 29 immune-related gene sets in different subtype samples.

### Differences in infiltrating immune cells in T2D

The abundance of immune cell infiltration based on the T2D gene expression dataset was calculated using the CIBERSORT^30^. The samples were filtered according to a *P*-value < 0.05. Next, an immune cell infiltration matrix was obtained. The difference in immune cell infiltration was visualized as violin plots. The correlation between the identified genes and infiltrating immune cells was analyzed.

### Construction of IR-DEGs–microRNA network and IR-DEGs–transcription factor (TF) network and identification of potential drugs interacting with IR-DEGs

We obtained miRNA and TF target genes using the NetworkAnalyst Database (https://www.networkanalyst.ca/), which integrates the miRNA Database (miRTarBase and TarBase Database) and TF Database (ENCODE). The IR-DEGs–miRNA and IR-DEGs–TF networks were visualized using Cytoscape. The drugs or molecular compounds that may interact with IR-DEGs were predicted using DGIdb (https://www.dgidb.org), and the drug–gene interaction networks were visualized using Cytoscape.

### Statistical analysis

The R program (https://www.r-projec t.org/, version 3.6.3) was used for all data and statistical analyses. For comparison of continuous variables with two groups, the statistical significance of the normally distributed variables was assessed via an independent Student t-test. Differences between non-normally distributed variables were compared via the Mann–Whitney U test (i.e., Wilcoxon rank sum test). P-values were two-sided, and data with a p-value < 0.05 were regarded as statistically significant.

### Ethics approval and consent to participate

All studies were performed in accordance with the approved guidelines of the Research Ethics committee of the Second hospital of Hebei Medical University (approval number: 2022-AE211).

## Results

**Figure 1:**
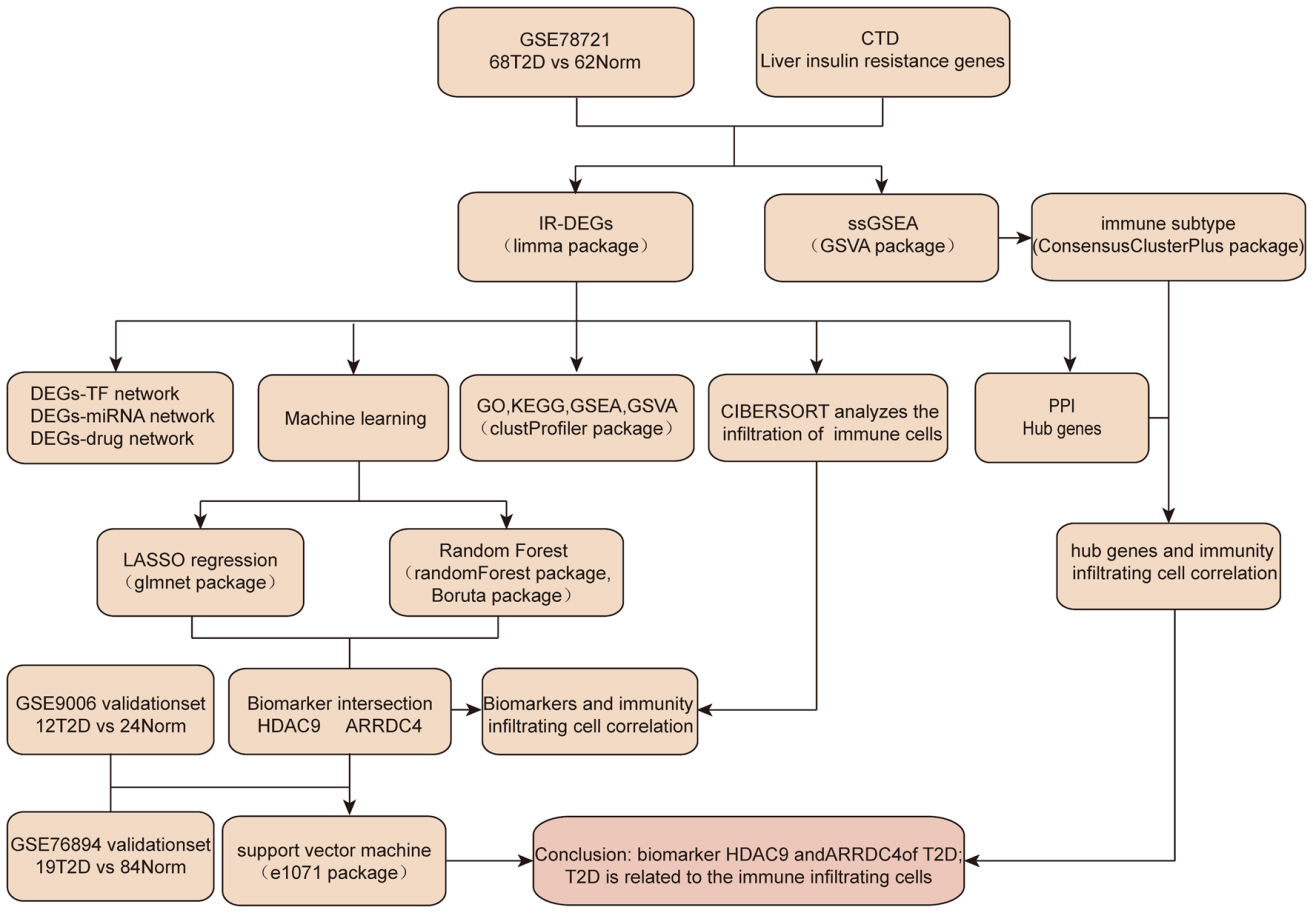
Flow chart of this study.

A flow diagram of the overall design of this study is presented in Fig. 1.

### Correlation Analysis and Identification of Immune Gene Set in T2D

**Figure 2:**
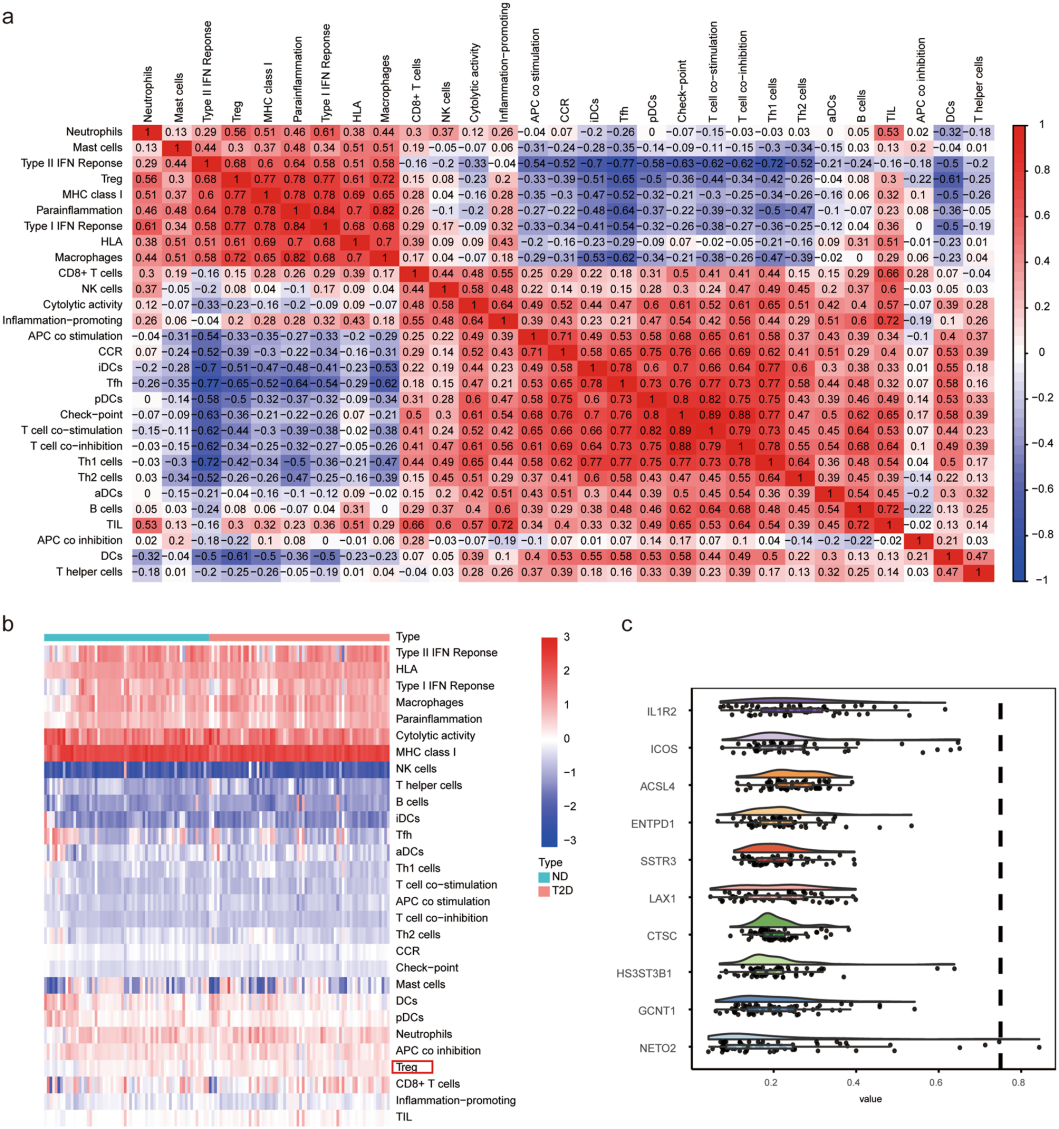
Correlation Analysis and Identification of Immune Gene Set in T2D. (**a**) The correlation heatmap of 29 immune-related gene sets in the T2D samples. The color of the colored square represents the strength of the correlation, with positive correlations in red and negative correlations in blue. The darker the colour, the stronger the correlation. (**b**) Heatmap shows the estimation of 29 immune-related gene sets in all samples. Each column indicates a sample, and each row indicates an immune gene set. Red represents high relative expression levels, blue represents low relative expression levels, the green annotation bar represents the T2D sample, and the red annotation bar represents the normal sample. Among them, Treg indicates the differential immune gene set. (**c**) The cloud and rain map of genes in the differential immune gene set based on functional similarity. Semantic similarity of Gene Ontology (GO) terms was calculated using the R package GOSemSim. The horizontal axis is the level of similarity, and the vertical axis is genes. Dot size represents the magnitude of correlation.

As shown in Fig. 2, the type I interferon (IFN) response positively correlated with major histocompatibility complex class I, Treg, and parainflammation. Th1 cells displayed a significant and positive correlation with T-cell co-inhibition, intelligent dendritic cells (iDCs), T follicular helper cells (Tfh), plastmacytoid dendritic cells (pDCs), and T-cell co-stimulation. Furthermore, the type II IFN response negatively correlated with iDCs, Tfh, and Th1 cells, and Tfh negatively correlated with Treg, parainflammation and macrophages (Fig. 2a). We identified a differential immune gene set–Treg by comparing ssGSEA scores of T2D samples with those of normal samples (Fig. 2b). In addition, we ranked the top 10 genes in the differential immune gene set based on functional similarity and found that *IL1R2, ICOS,* and *ACSL4* were the top three genes (Fig. 2c).

### T2D immune subtype identification

**Figure 3:**
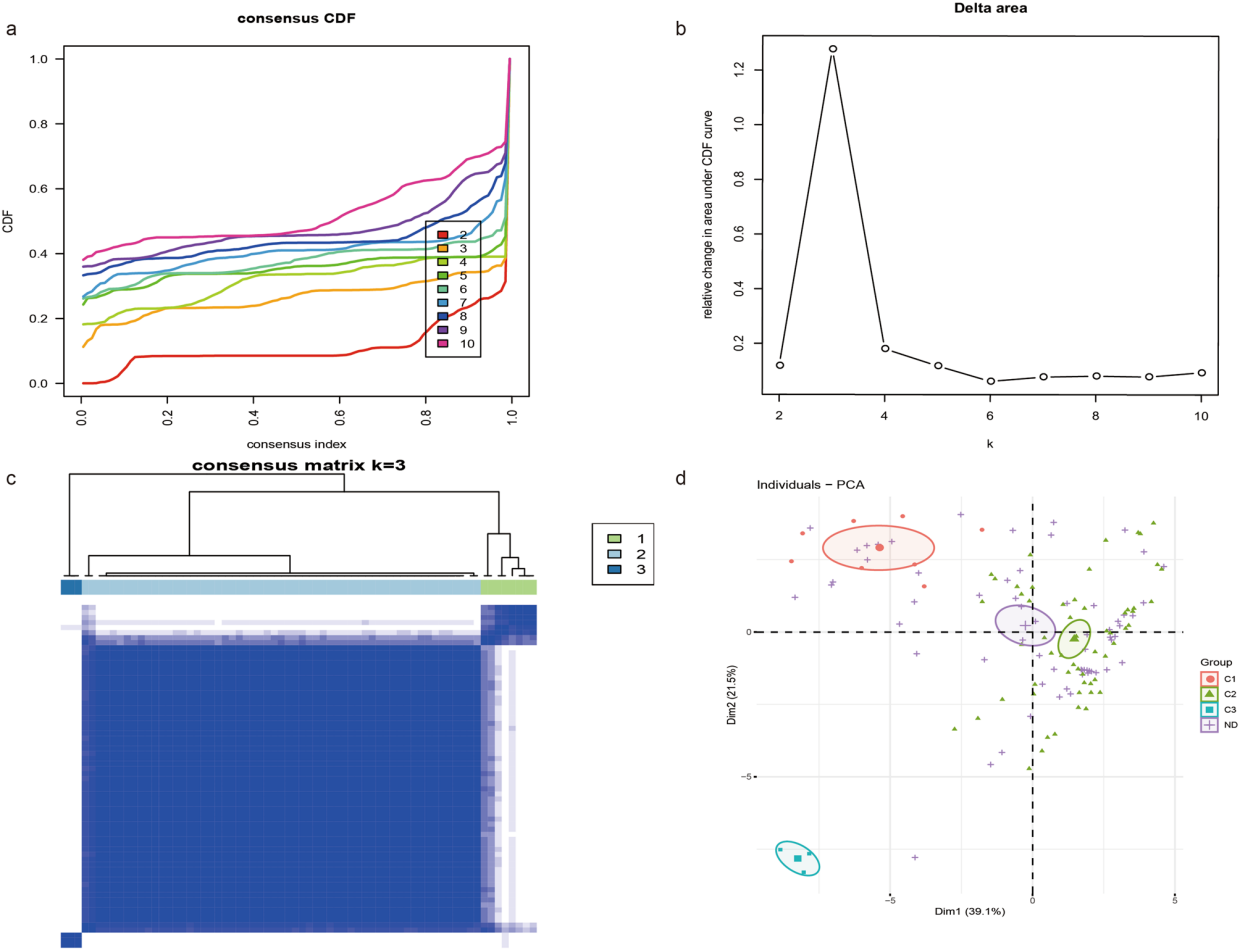
Consistent clustering of T2D samples. (**a**) Consensus clustering cumulative distribution function (CDF) for k = 2–10. (**b**) Relative change in area under CDF curve for K = 2–10. (**c**) Consensus clustering matrix for k = 3. A higher consensus score between two samples indicates that they are more likely to be assigned to the same cluster in different iterations. (**d**) Principal component analysis (PCA) of all samples based on the ssGSEA score. The cross represents normal, the dot represents subtype 1, the triangle represents subtype 2, and the square represents subtype 3.

The molecular classification of T2D was identified using unsupervised consensus clustering of all T2D samples to reveal the diverse immune cell population infiltration in T2D. The value of K determined the optimality number of clusters. The “ConsensusClusterPlus” package was executed to split T2D samples into k subtypes (k = 2–10). According to the relative change in the area under the CDF curve and consensus heatmap, the optimum was k = 3, and no apparent augment was displayed in the area under the CDF curve (Fig. 3a–c). In addition, PCA indicated that T2D samples could be divided into three subtypes (Fig. 3d).

### Function and pathway enrichment analysis of IR-DEGs

We employed R software to extract 2239 DEGs from the GSE78721 gene expression data and established the intersection of the hepatic IR genes from CTD to obtain 2094 IR-DEGs (Supplementary Table 2). We first performed GO functional enrichment analysis on 2094 IR-DEGs, and the results were as follows (Supplementary Table 3): IR-DEGs were particularly enriched in regulation of lipid metabolic process, response to endoplasmic reticulum stress, and the toll-like receptor signaling pathway in the biological process category. Moreover, IR-DEGs were significantly enriched in multiple important KEGG pathways (Supplementary Table 3), including spliceosome, MAPK signaling pathway, TNF signaling pathway, and AMPK signaling pathway. GSEA–GO enrichment analysis indicated that the cellular macromolecule metabolic process, positive regulation of biological process, and cellular response to stimulus were primarily enriched in the biological process category. GSEA–KEGG enrichment analysis indicated that the spliceosome, lysosome, and other pathways were significantly enriched (Supplementary Table 4). Furthermore, we performed GSVA with the MsigDB gene sets; six hallmarks were differentially enriched between normal tissues and T2D tissues (Supplementary Table 5).

### Construction of IR-DEGs–miRNA network and IR-DEGs–TF network and identification of potential drugs of IR-DEGs

The top three miRNA-target IR-DEGs included *NUFIP2, CCND2,* and *ZNF264,* which were targeted by 538 miRNAs, 363 miRNAs, and 347 miRNAs, respectively. Importantly, hsa-mir-1-3p was identified as the miRNA that may control the largest number of IR-DEGs (Supplementary Fig. 2a, d). The top five target IR-DEGs of TF were *ACBD4,* modulated by 149 TFs; *BAX,* modulated by 126 TFs; *CCDC142,* modulated by 122 TFs; *AKT1S1,* modulated by 101 TFs; and *HES4,* modulated by 97 TFs (Supplementary Fig. 2b, e). A total of 36 drugs or molecules such as melphalan, cabazitaxel, and azathioprine were found to interact with *CYP3A4*. Moreover, 29 drugs or molecules (including everolimus) regulated *KRAS*, and 21 drugs or molecules (including dexamethasone and leflunomide) regulated *NFE2L2* (Supplementary Fig. 2c, f. Supplementary Table 6–7).

### Selection and validation of biomarkers for T2D using machine learning approaches

LASSO and RFB analyses were combined to identify candidate genes for T2D diagnosis. Seventeen and sixteen genes were determined, respectively (Supplementary Fig. 3a–d). Two key genes, *HDAC9* and *ARRDC4*, shared by the two feature selection algorithms were identified as diagnostic markers (Supplementary Fig. 3e). To evaluate their effectiveness of diagnosing, we used SVM model and “pROC” package. The AUC of the SVM model in the GSE78721 training set was 71.0%, demonstrating that the model could accurately distinguish between T2D and normal samples (Supplementary Fig. 3f).

**Figure 4:**
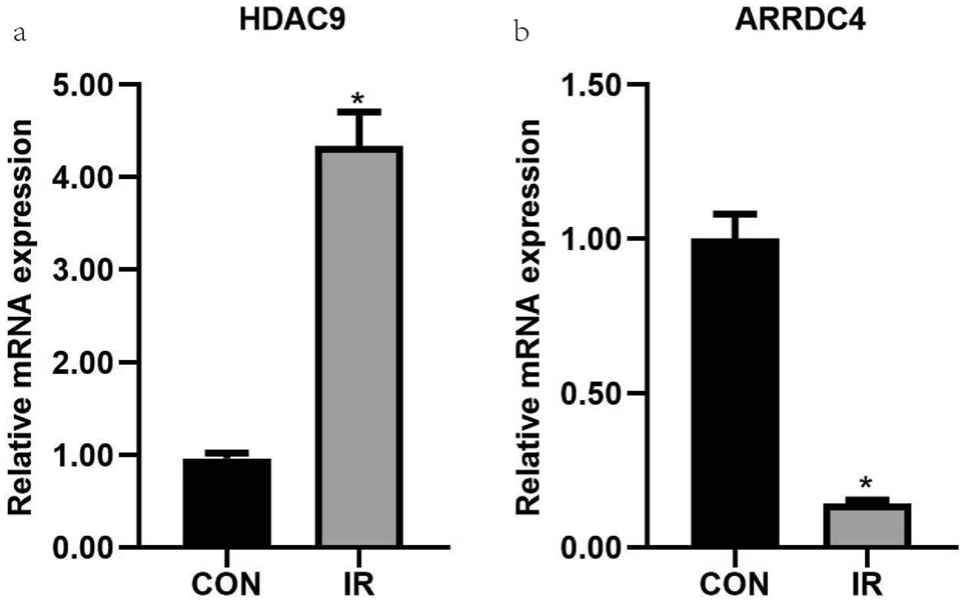
Verification of the biomarkers. Total RNA was extracted from the hepatic tissues of insulin resistance mice and control mice for qRT-PCR analysis. The expression of ARRDC4, and HDAC9 in the mice (n = 6 per group). *P* < 0.05 versus the control. The data are presented as the mean ± SD.

Then, the GSE76894 and GSE9006 validation datasets were used to assess the model performance and avoid overfitting to the training set. The AUC was 72.7% and 81.9%, respectively (Supplementary Fig. 3g–h). We further validated the reliability of the results by measuring the biomarkers expression levels in the hepatic tissues of IR mice using qRT-PCR. After 8 weeks different diets, the blood glucose and insulin levels of mice in the IR group were higher than those in the control group, while the QUICKI levels were opposite, which confirmed the successful establishment of insulin resistance model mice (Supplementary Table 8). *HDAC9* mRNA level was elevated in HFD-fed mice compared with those in normal mice, *ARRDC4* showed the opposite trend (Fig. 4). Thus, *HDAC9* and *ARRDC4* could be considered accurate and efficient biomarkers for T2D diagnosis.

### Visualization of PPI and hub genes related to IR-DEGs

**Figure 5:**
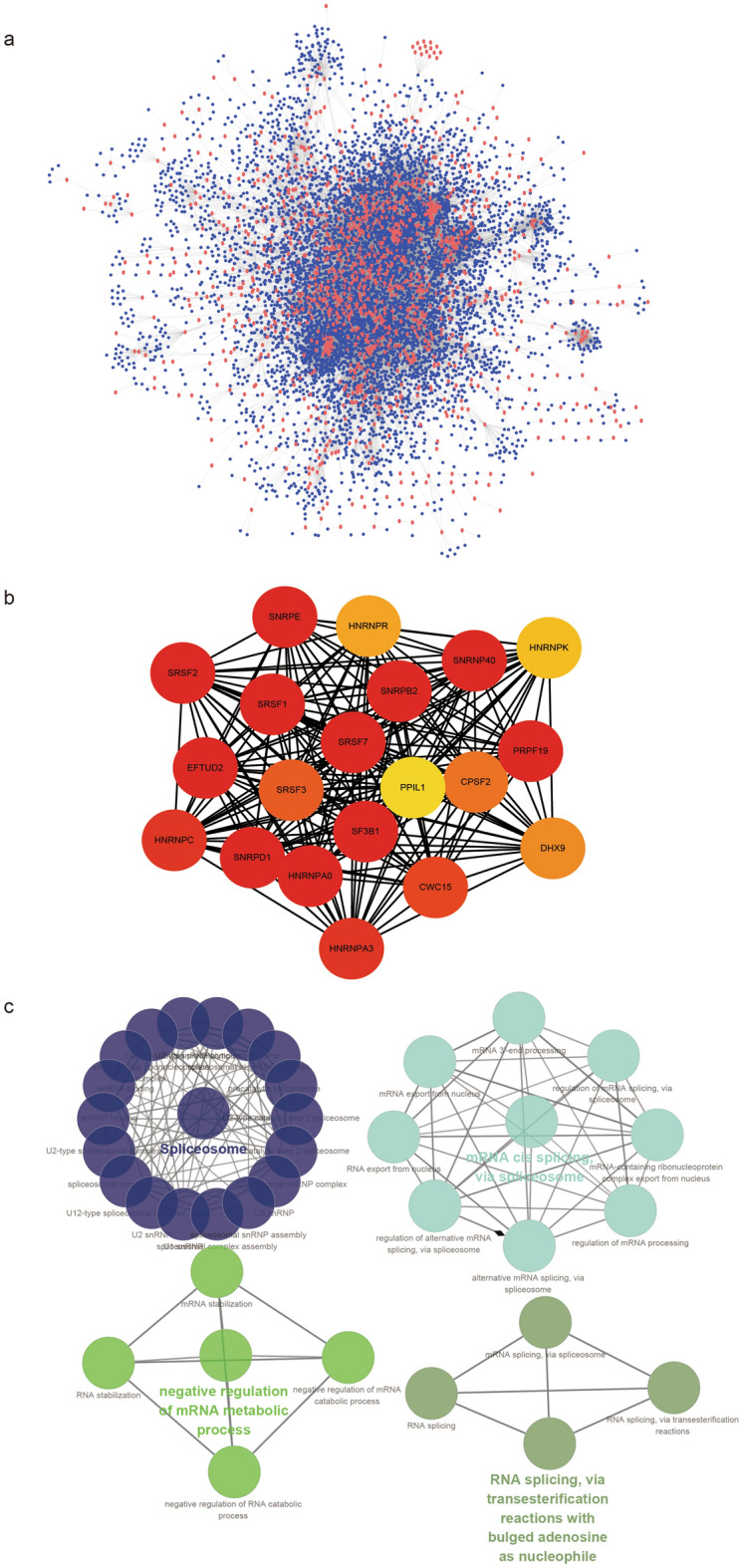
Visualization of protein–protein interaction (PPI) networks and hub-genes. (**a**) The PPI network of IR-DEGs, the red nodes represent IR-DEGs. Red nodes represent IR-DEGs and blue nodes represent IR-DEGs-related genes. (**b**) Hub genes were identified from the PPI network using the maximum group centrality (MCC) algorithm, and the nodes represent 20 hub genes. (**c**)The biological function annotation analysis of the selected hub genes were performed by ClueGO and CluePedia.

We used the PPI information in the STRING database to extract the IR-DEGs related to the PPI network (Fig. 5a), and identified 20 hub genes (Fig. 5b). Figure 5c illustrates that hub genes were primarily enriched in spliceosome, negative regulation of the mRNA metabolic process, mRNA cis splicing via spliceosome, RNA splicing, and via transesterification reactions with bulged adenosine as a nucleophile among others.

### Correlation analysis of immune infiltration and immune related genes

**Figure 6:**
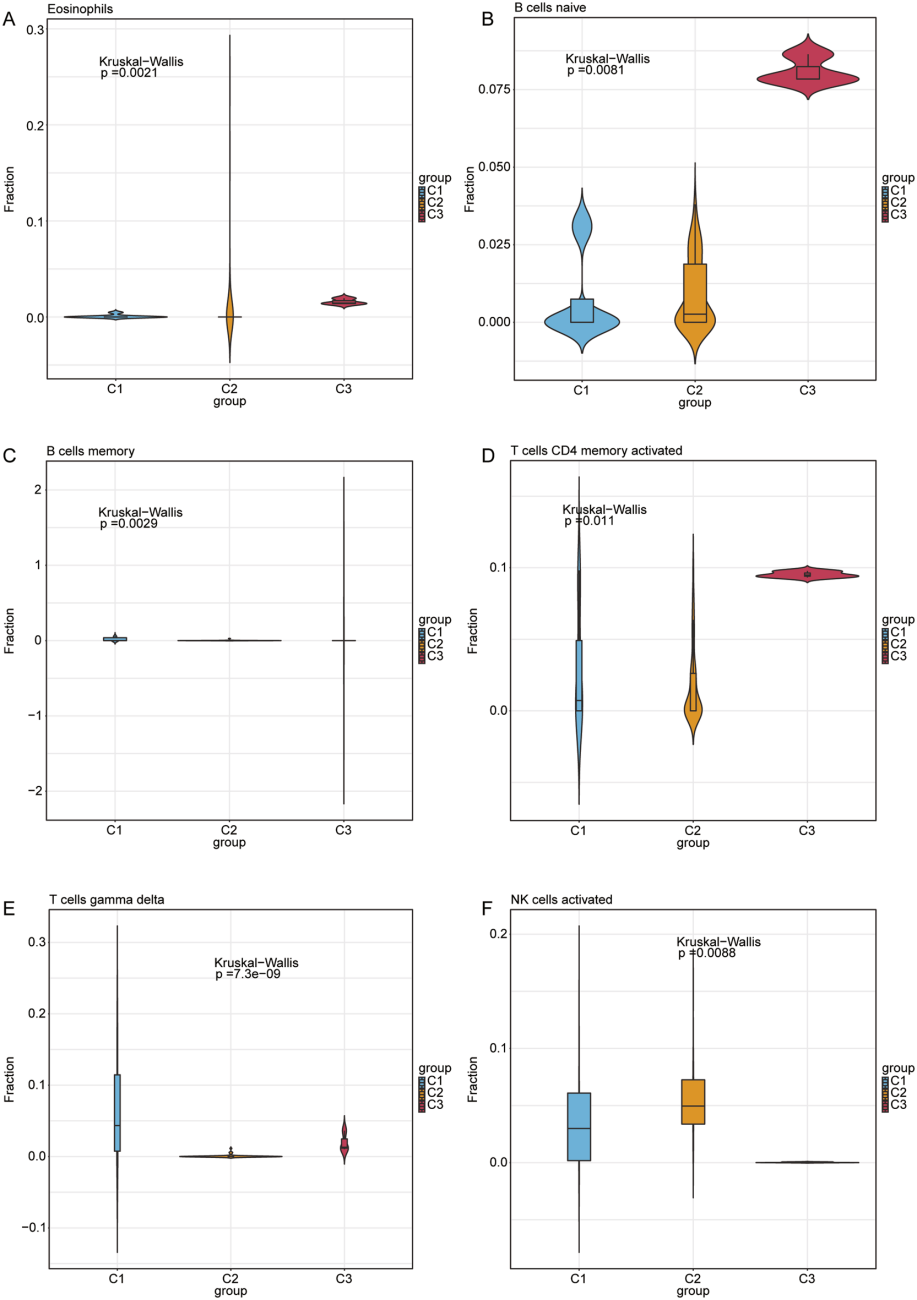

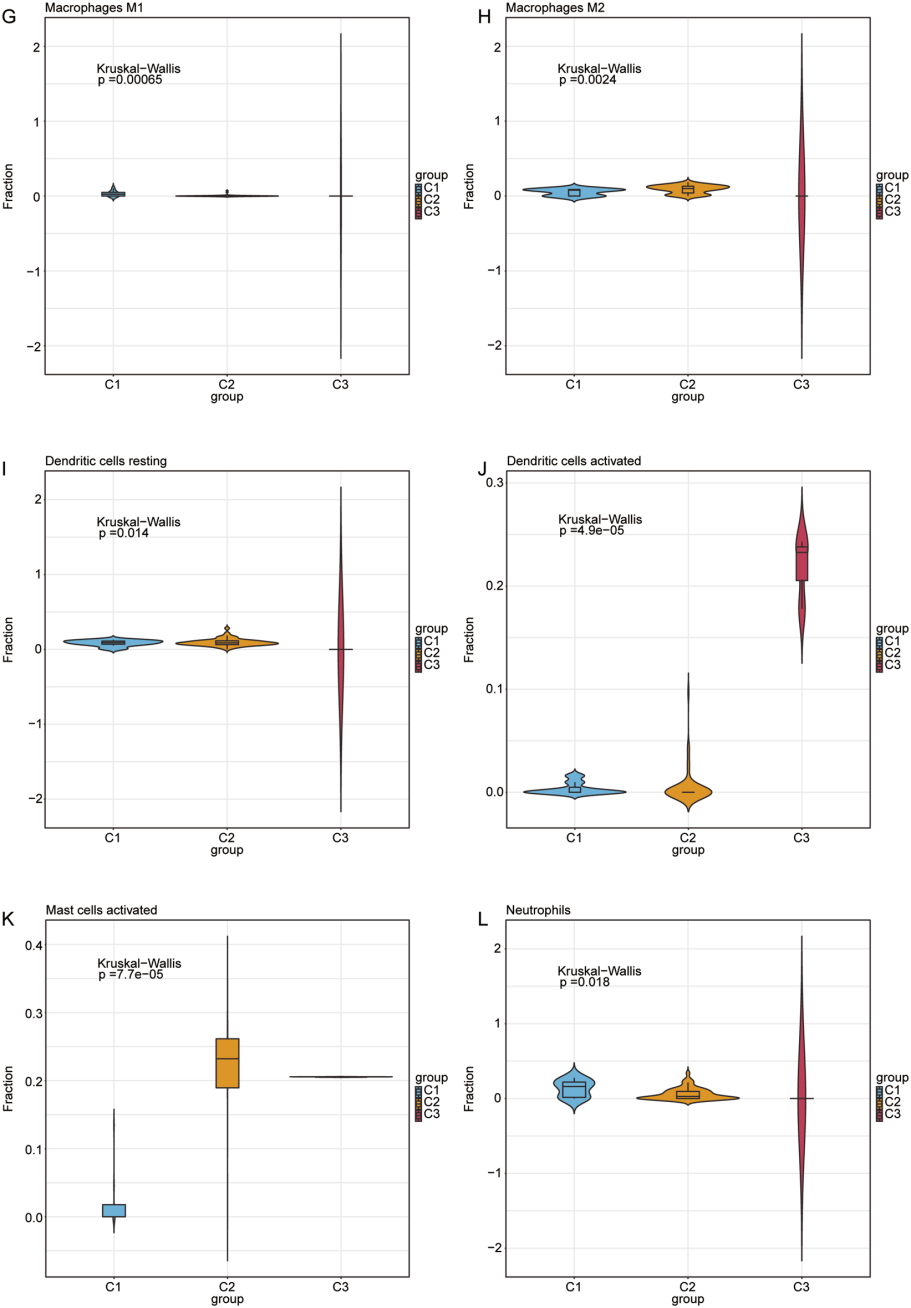
Evaluation and visualization of immune cell infiltration in three immune characteristic T2D subtypes. (**a**–l) Violin plots of immune cells that are significantly different between the C1, C2, and C3 subtypes. The blue is the C1 subtype, the orange is the C2 subtype, and the red is the C3 subtype.

Binding of the spliceosome protein Eftud2 to the elongation factor Tu GTP is an AS mechanism that may regulate the innate immune response of macrophages^31^. *SRSF1, SRSF3, SRSF5,* and *SRSF9* act as common splicing factors and are major classical SR proteins^32^. *SRSF1* involved in transcription activation, regulated AS and mRNA nuclear export in the immune system^33^. Moreover, SRSF1 interacts with a short sequence within CD46 exon 13 to produce the CYT2 isoform^34^ , which is crucial for regulating T-cell differentiation and homeostasis. Increasing evidence has confirmed that *SRSF2* regulated splicing variants related to cancer and immune disorders^35,36^. The box plot in Fig. 6a shows that *HNRNPA0, EFTUD2, PRPF19, SRSF2, SRSF7, SF3B1, SNRPE, SNRNP40, SNRPB2, SRSF1, HNRNPC, HNRNPA3, SRSF3, CPSF2, HNRNPR, HNRNPK,* and *PPIL1* had significant differences in the three immune characteristic T2D subtypes. The correlation heatmap indicates differences between 20 hub genes and 29 immune-related gene sets in the three immune characteristic T2D subtype samples (Supplementary Fig. 4b–d). In the C1 subtype, *SRSF3* and *SRSF7* were negatively correlated with the immune gene sets of B cells and T helper cells and were positively correlated with immune gene sets of the Type I IFN response (Supplementary Fig. 4b). In the C2 subtype, *SRSF3* and *SRSF7* were positively correlated with the immune gene sets of Th1, NK, and Th2 cells, and negatively correlated with the immune gene sets of Type II IFN response, macrophages, and T-helper cells (Supplementary Fig. 4c). In the C3 subtype, *SRSF3, SRSF7* and other hub genes were negatively correlated to the immune gene sets of Tfh, HLAs, and pDCs. *SRSF3* and *SRSF7* were positively correlated with the immune gene sets of type I IFN response and parainflammation (Supplementary Fig. 4d).

### Immune cell infiltration analysis

The violin plots of the 22 immune cell infiltrations from CIBERSORT analysis indicate significant differences among the three immune characteristic T2D subtype samples: eosinophils, B cells naive, B cells memory, T-cells CD4 memory activated, T-cells gamma delta, NK cells activated, macrophages M1, macrophages M2, dendritic cells resting, dendritic cells activated, mast cells activated, and neutrophils infiltration (Fig. 6a–l). Correlation analysis revealed that *HDAC9* exhibited a positive correlation with naive B cells (r = 0.204, *p* = 0.020) and a negative correlation with plasma cells (r = − 0.257, *p* = 0.003) and resting mast cells (r = − 0.204, *p* = 0.020) (Supplementary Fig. 5a). *ARRDC4* correlated positively with NK cells activated (r = 0.306, *p* = 0.0004), macrophages M2 (r = 0.440, *p* = 1.65e-07), and activated mast cells (r = 0.474, *p* = 1.20e-08) and negatively with B cells memory (r = − 0.480, *p* = 7.44e-09), T-cells CD8 (r = − 0.295, *p* = 0.0007), Tregs (r = − 0.266, *p* = 0.002), T-cells gamma delta (r = − 0.503, *p* = 1.08e-09), monocytes (r = − 0.256, *p* = 0.003), and macrophages M1 (r = − 0.435, *p* = 2.29e-07) (Supplementary Fig. 5b).

## Discussion

Although previous studies have explored T2D mRNA expression profiles in the GEO database^37–39^, few studies have combined the CTD database with GEO database to analyze the possible role of hepatic IR genes and immune cell infiltration in T2D^40,41^. Hence, in this study, *HDAC9* and *ARRDC4* were identified as potentially accurate and efficient biomarkers for T2D diagnosis. Twenty hub genes were identified, whose expressions correlate with immune cell infiltration in T2D. In this study, bioinformatic quality control was done including microarray data quality assessment, normalization and batch effects correction, and cross validation.

Among the 105 BP annotations, the regulation of lipid metabolic processes, response to endoplasmic reticulum stress, and the toll-like receptor signaling pathway were considered significantly associated with IR. Excessive free fatty acid storage in muscles, the liver, and adipose tissue causes IR, leading to aggravation of lipid metabolism disorder, forming a vicious cycle^42^. Activating protein kinase R-like endoplasmic reticulum including inositol-requiring kinase 1 and X box binding protein splicing increases NF-κb, TNF-α, and IL-6 level, leading to the phosphorylation of related receptors, which in turn affects insulin signaling pathways^43^. Moreover, Tian et al^44^ found that T2D mice had increased TLR2 level in their skeletal muscle cells compared with normal mice. The knockout of the TLR4 gene, blocking TLR4-mediated inflammatory signaling, increased the glucose tolerance and sensitivity to insulin, and activation of the TLR4 signaling pathway caused the body to be in a state of low-grade inflammation, which is an important factor in IR^45^ .

Further, among 62 enriched signaling pathways, the MAPK, TNF, and AMPK signaling pathways were related to IR. Notably, inhibiting p38 MAPK activity ameliorates liver insulin sensitivity in obesity-associated disorders^46^. p38 MAPK activation induces the phosphorylation of insulin receptor substrate 1(IRS1) inhibition, which results in inhibiting insulin signaling^47^. Patients with T2D have increased levels of TNF-α^48^. TNF-α reduction enhanced insulin-induced Akt phosphorylation and ameliorated lipid-induced IR in diabetic hepatocytes^49^. Previous studies have indicated that AMPK activation could further promote IRS1 phosphorylation downstream of the insulin signaling pathway and activate Akt in driving the translocation of GLUT4 to the cell membrane, thereby promoting the uptake and utilization of glucose and ultimately contributing to attenuating IR^50,51^.

Previous research has demonstrated that miRNA modulates islet β cell function or IR in T2D humans, and could be regarded as a potential target for the early diagnosis and effective treatment of T2D^52^. For example, in obese mice, miR-103/107 expression is elevated^53^. Silencing of miR-103/107 improves protein levels of the insulin receptor regulator pavement protein-1, thereby affecting glucose homeostasis. A recent study showed that miR-30b expression was significantly elevated in the liver of rats fed HFD^54^. miR-30b activates endoplasmic reticulum stress. Activation of hepatic endoplasmic reticulum stress impairs insulin sensitivity in rats promoting the insulin signalling pathway and inhibiting hepatic adipogenesis. Studies have shown that miRNA could directly target and regulate the expression of multiple target mRNAs and their protein expression in the process of IR, and the target mRNA encoding the protein can also be regulated by multiple miRNAs^55,56^.Our findings are consistent with those of previous studies; the miRNA that may participate in the regulation of the largest number of IR-DEGs was hsa-mir-1-3p.

TFs can affect cell metabolism, forkhead box 3a TFs regulate the expression level of Bax in the process of diabetic cardiomyocyte apoptosis^57^. Transcription factors can affect cell metabolism through transcription, translation, and encoding. Previous studies have shown that transcription factors regulate the expression level of Bax in the process of diabetic cardiomyocyte apoptosis^57^, and also play a role in the apoptosis of diabetic podocytes^58^. The present study showed the multiple transcription factors associated with IR in the network.

The drug-target network indicated that melphalan, cabazitaxel, and azathioprine were found to interact with CYP3A4, a critical enzyme involved in the hepatic metabolism of repaglinide (oral hypoglycemic drug)^59^. Growing evidence has indicated that diabetes mellitus could cause marked alterations in the CYP3A4 activity and expression^60^ and elevated fatty acids levels lead to enhanced CYP3A4 function and expression^61^. This may provide new evidence in the clinical search for effective drugs to treat diabetes.

Recently, T2D has been identified as an immune-related inflammatory disease, and abnormal immune regulation is strongly related to T2D pathogenesis^62^. In this study, we identified a differential immune gene set–Tregs by comparing ssGSEA scores of T2D samples with those of normal samples, which are the main controllers of the body's immune tolerance and are associated with long-term immune homeostasis and tissue inflammation^63^. Recent studies have shown that Tregs may mediate metabolic diseases, including atherosclerosis^64^, obesity^65^, and T2D^66^. The imbalance of Th1/Th2 and the decrease in Treg level in patients with T2D indicated that abnormal.

T-cell subsets exist in T2D^66^. Yuan and colleagues^67^ discovered that the proportion of Tregs was significantly reduced and was negatively correlated with IR individuals with newly diagnosed T2D. We discovered that the immune-related genes *IL1R2, ICOS, ACSL4* could participate in regulating Tregs in T2D. However, the underlying mechanisms require further investigation using cell and animal model experiments.

In this study, we identified two IR-related genes (*HDAC9* and *ARRDC4*) closely related to T2D. It has been suggested that *HDAC9*, which is involved in the generation of reactive oxygen species, apoptosis, and inflammation modulates adipogenic differentiation and hepatic insulin sensitivity^68,69^. *HDAC9* expression was found to be upregulated in skeletal muscle tissue of IR women with metabolic syndrome^70^. In vivo and in vitro experiments in diabetic nephropathy models have demonstrated that *HDAC9* can induce podocyte apoptosis and renal injury through the JAK2/STAT3 pathway^71^. *HDAC9* induces the expression of phosphoenolpyruvate carboxykinase and glucose 6-phosphatase through deacetylation^72^. It was found the visceral adipose tissue (VAT) mRNA expression level of HDAC9 were significantly lower in obese patients in comparison with normal-weight women. The VAT HDAC9 mRNA level inversely correlated with BMI, waist, hip and with HOMA-IR, insulin levels, and serum concentration of hs-CRP^73^. In contrast, Chatterjee et al. found that HDAC9 expression increased in adipocytes of diet-induced obesity mice in comparison with chow-fed mice. HDAC9 gene deletion improves adipogenic differentiation and systemic metabolic state during an HFD, resulting in improved glucose tolerance and insulin sensitivity, and prevented hepatosteatosis^68^. In addition, a gene expression microarray analysis identified that HDAC9 was significantly correlated with insulin resistance^74^. *ARRDC4* is associated with innate immunity^75^ ; however, the relationship between *ARRDC4* and IR is not yet clear. Our findings suggested that these two-genes are important biomarkers affecting the diagnosis of T2D. To better understand *HDAC9*-related inflammatory activities, the present study revealed that *HDAC9* was positively correlated with B cells but negatively correlated with the plasma cells and mast cells when resting; we further validated the values of *HDAC9* and *ARRDC4* as T2D markers using qRT-PCR. Expression of *HDAC9* and *ARRDC4* are useful in differentiating T2D from normal tissue. However, the underlying mechanisms need to be further analyzed in future studies.

Our study has some limitations. First, western blot, immunohistochemistry approaches, and other tools should be used to further determine the regulatory effect of hub genes of T2D. Second, to determine the effects of DEGs in T2D, it remains essential to conduct concrete double gain and loss-of-function experiments. Third, we have not identified any data on immunotherapy for type 2 diabetes in any public databases, and we would collect patient samples of immunotherapy and control samples for sequencing in further study. Fourth, although we assessed the immune infiltration-related signature of T2D, a detailed functional analysis is necessary to discover potential immunomodulatory mechanisms. Finally, in our study, we used a microarray gene expression dataset of type 2 diabetes patients to identify potential biomarkers. However, it is worth noting that we only used animal studies during the validation phase. Although these results provide us with initial insights, additional validation studies, particularly on human samples, are needed before these biomarkers can be used in the clinic. In our study, we did not validate the biomarkers by integrating multiple GEO datasets, which may have introduced data bias to some extent. We intend to consider more GEO datasets in subsequent studies.

## Conclusions

Overall, this study identified two key biomarkers (*HDAC9* and *ARRDC4*) and 20 hub genes, which could be used as novel potential biomarkers and therapeutic targets for T2D. Meanwhile, the immune cell infiltration in the T2D subtype and the relationship between 20 hub genes and 29 immune-related genes sets in the three different T2D subtype samples were analyzed providing new evidence of immunotherapy in T2D. These observations revealed new insights into the development of novel diagnosis and therapeutic strategies for T2D.

### Supplementary Information

Supplementary Figure 1.

Supplementary Figure 2.

Supplementary Figure 3.

Supplementary Figure 4.

Supplementary Figure 5.

Supplementary Tables.

Supplementary Legends.

## Data Availability

The datasets supporting the conclusions of this article are available in the Gene Expression Omnibus (GEO) datasets: https://www.ncbi.nlm.nih.gov/ Gene Expression Omnibus (GEO) GSE78721, https://www.ncbi.nlm.nih.gov/ Gene Expression Omnibus (GEO) GSE76894, and https://www.ncbi.nlm.nih.gov/ Gene Expression Omnibus (GEO) GSE9006.

## Abbreviations

IR: Insulin resistance
T2D: Type 2 diabetes
ssGSEA: Single sample gene set enrichment analysis
DEGs: Differentially expressed genes
GO: Gene ontology
KEGG: Kyoto Encyclopedia of Genes and Genomes
GSVA: Gene set variation analysis
Tregs: Regulatory T cells
GEO: Gene Expression Omnibus
CTD: Comparative Toxicology Genetics Database
CDF: Cumulative distribution function
PCA: Principal component analysis
DO: Disease ontology
miRNA: MicroRNA
TF: Transcription factor
DGIdb: Drug Gene Interactions Database
LASSO: Least absolute shrinkage and selection operator
RFB: Regression and random forest and boruta
ROC: Receiver operating characteristic
AUC: Area under the curve
SVM: Support vector machine
RBF: Radial basis function
PPIs: Protein–protein interactions
MCC: Maximal Clique Centrality
UPR: Unfold protein response
AS: Alternative splicing
ANOVA: A one-way analysis of variance
