# Supplementary figures and images for: Identification of HDAC9 and ARRDC4 as potential biomarkers and targets for treatment of type 2 diabetes
Source: Sci Rep. 2024 Mar 25;14:7083. doi: 10.1038/s41598-024-57794-5 (PMC10963792; doi:10.1038/s41598-024-57794-5)

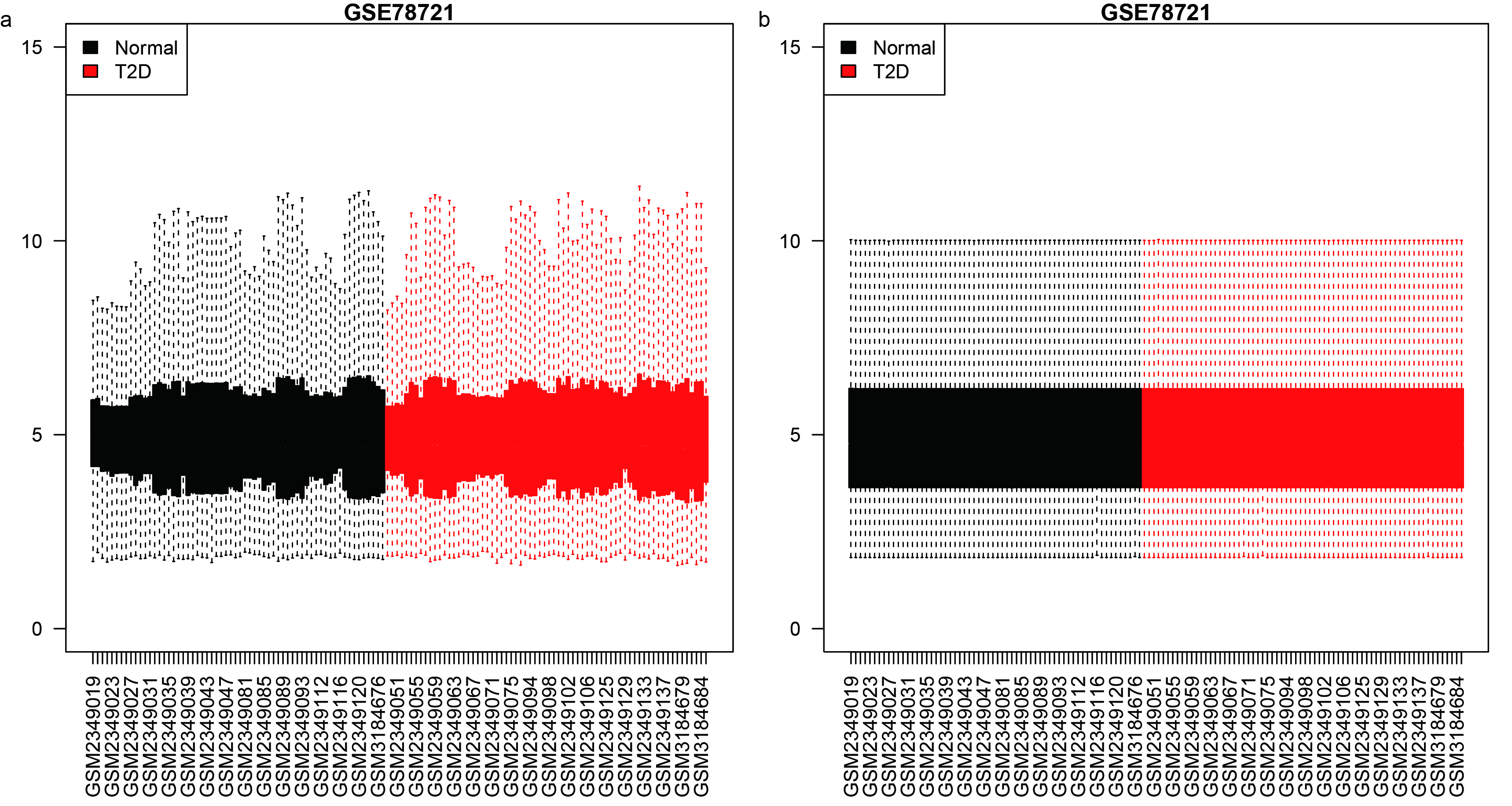

Supplement: Supplementary file 1 — Supplementary Figure 1. [file 41598_2024_57794_MOESM1_ESM.tif]

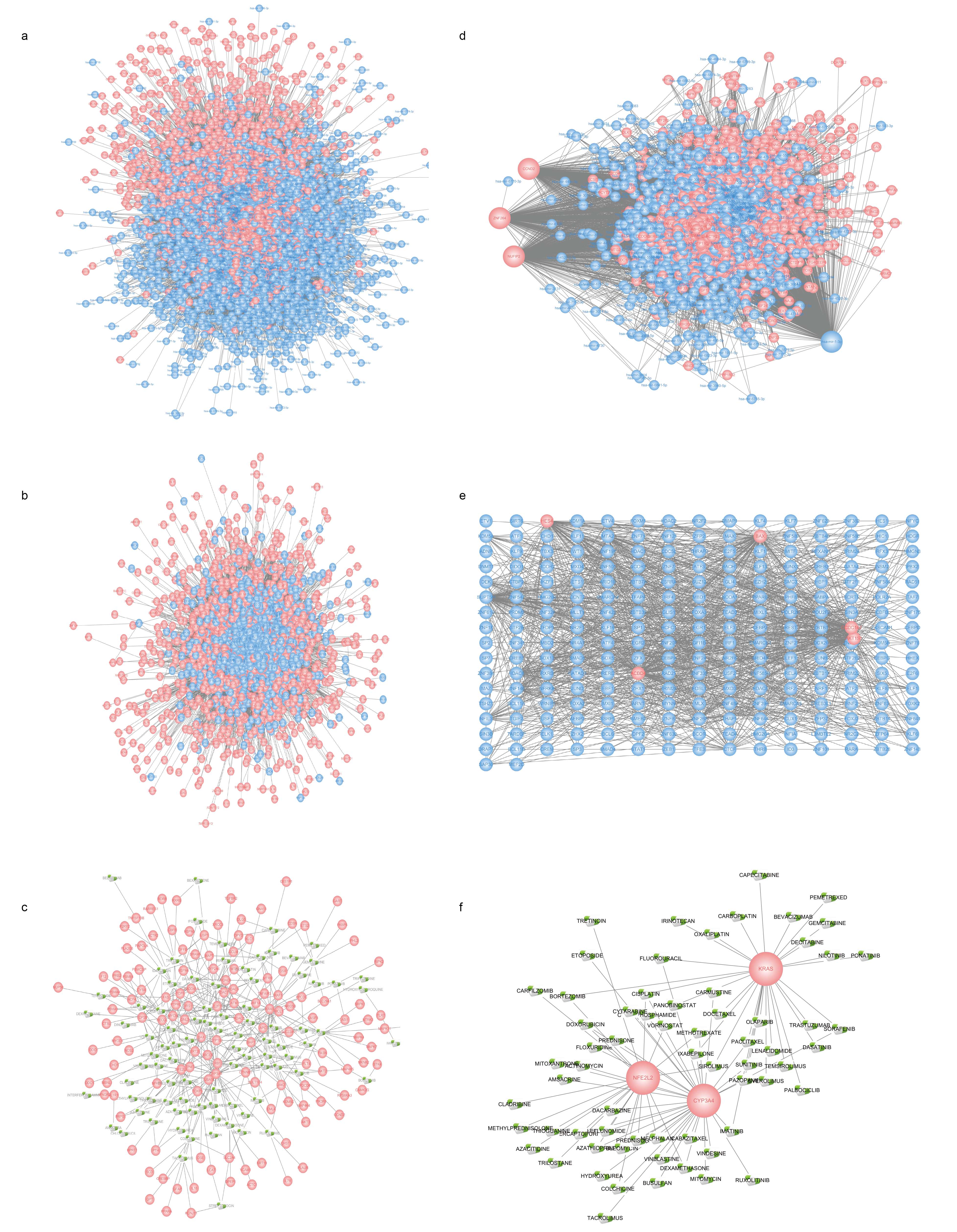

Supplement: Supplementary file 2 — Supplementary Figure 2. [file 41598_2024_57794_MOESM2_ESM.tif]

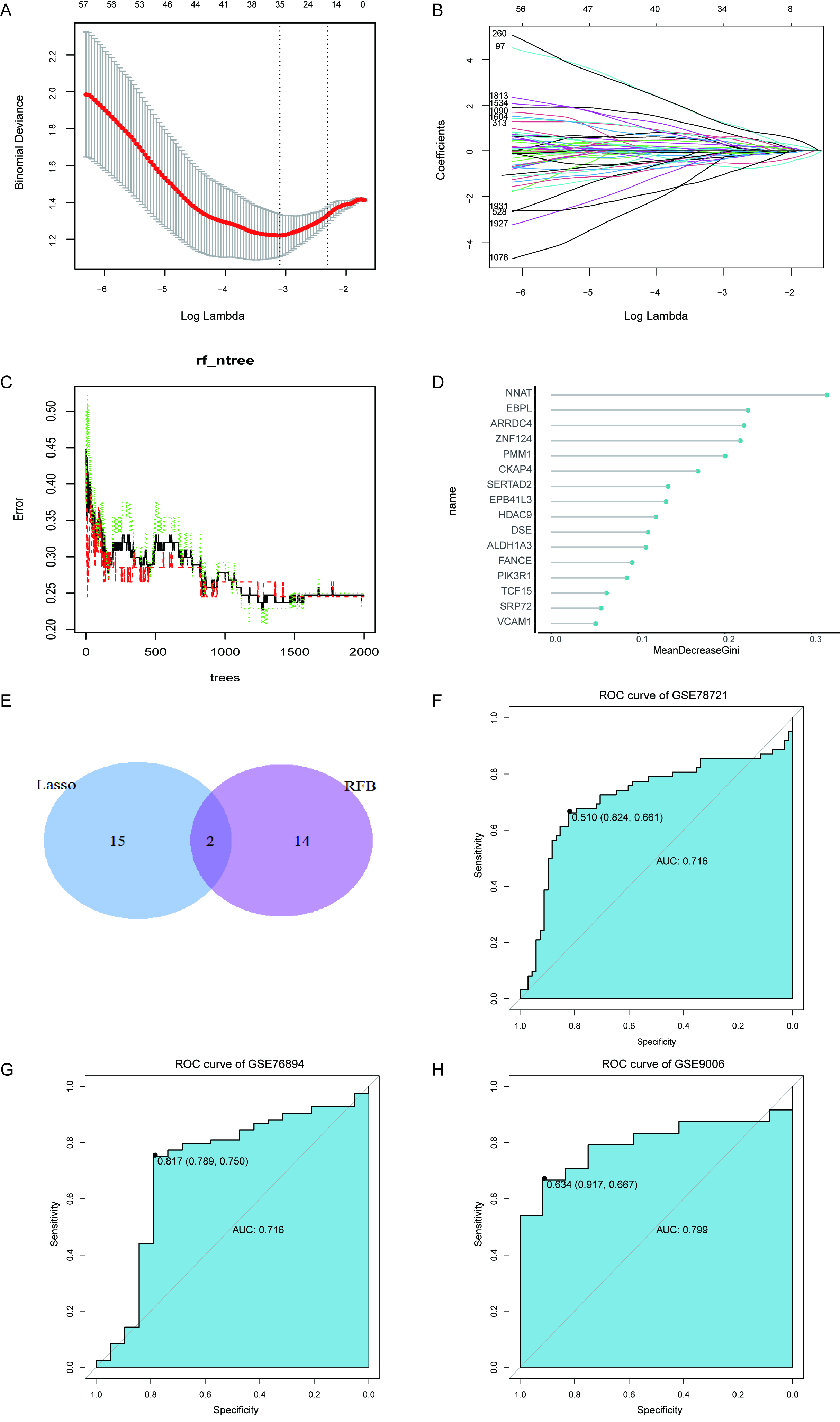

Supplement: Supplementary file 3 — Supplementary Figure 3. [file 41598_2024_57794_MOESM3_ESM.tif]

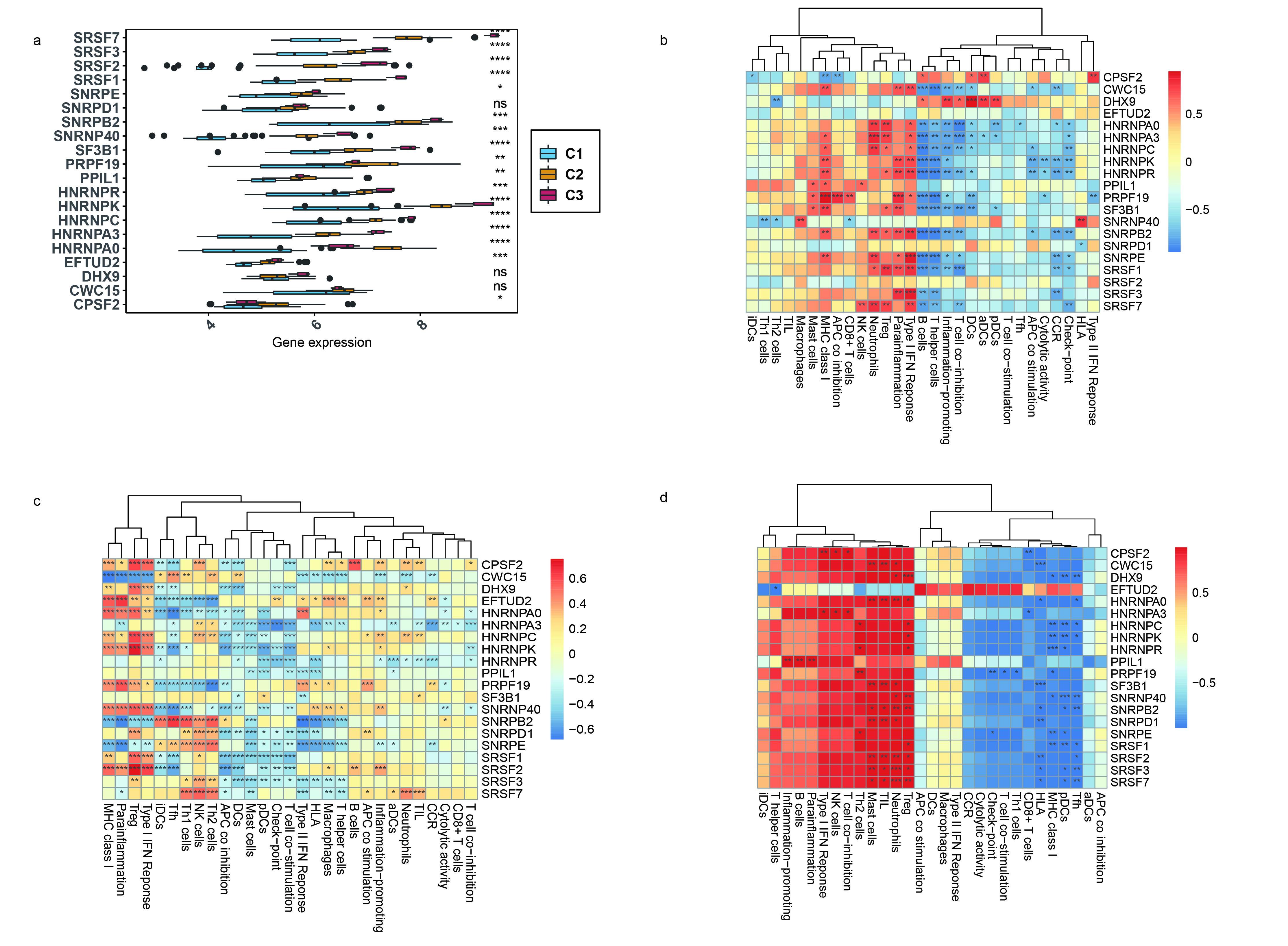

Supplement: Supplementary file 4 — Supplementary Figure 4. [file 41598_2024_57794_MOESM4_ESM.tif]

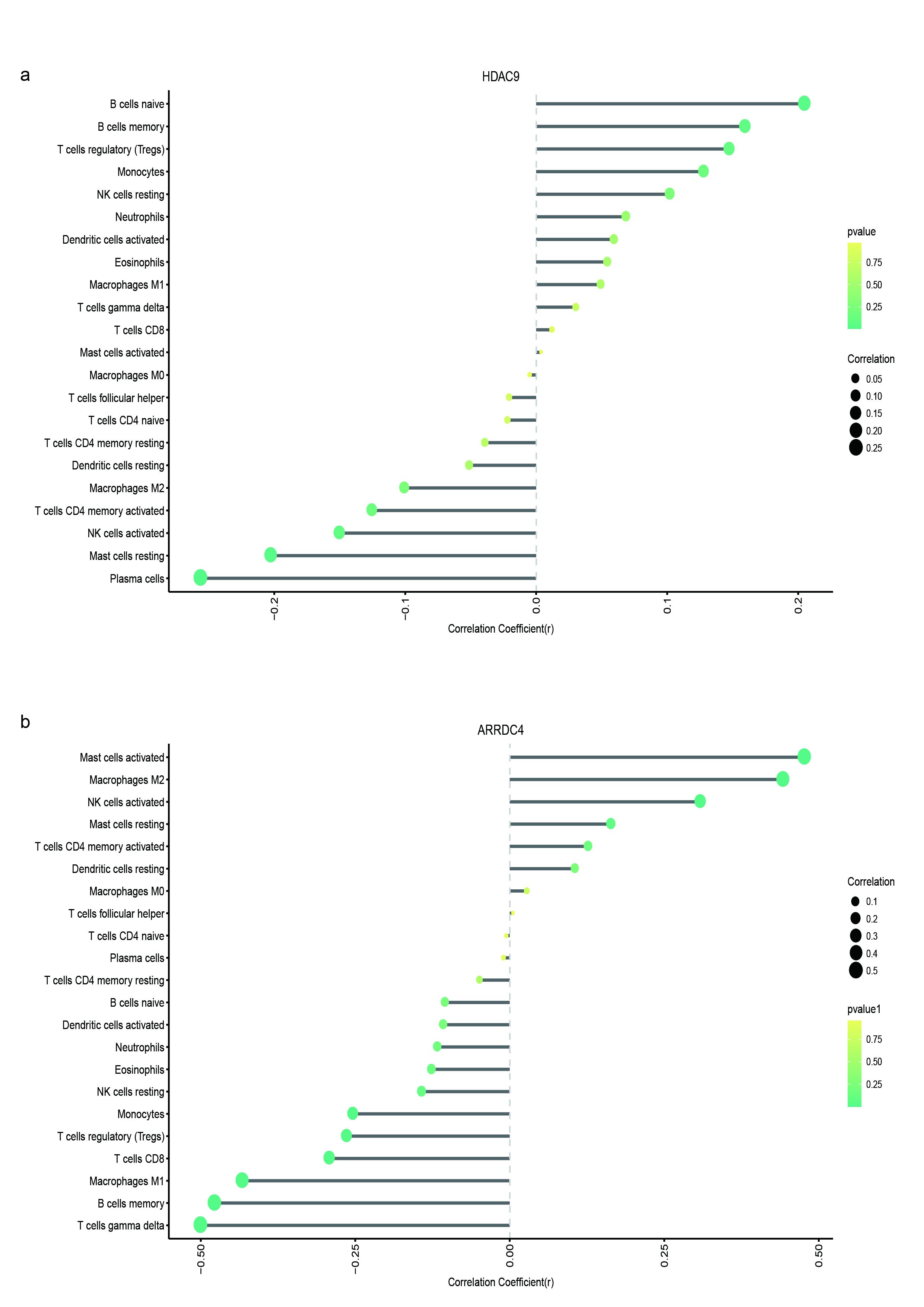

Supplement: Supplementary file 5 — Supplementary Figure 5. [file 41598_2024_57794_MOESM5_ESM.tif]
