# Supplementary material for: Identification of HDAC9 and ARRDC4 as potential biomarkers and targets for treatment of type 2 diabetes
Source: Sci Rep. 2024 Mar 25;14:7083. doi: 10.1038/s41598-024-57794-5 (PMC10963792; doi:10.1038/s41598-024-57794-5)
Supplement: Supplementary file 7 — Supplementary Legends. [file 41598_2024_57794_MOESM7_ESM.pdf]

Legends of supplementary figures and tables

Supplementary Fig. 1 Batch effects of the GSE78721 dataset.

Supplementary Fig. 2 Target gene–miRNA network and target gene–TF network and target gene–drug network. (a) Target gene–miRNA network, the nodes in the orange circle are genes, and the nodes in the blue circle are miRNAs. (b) Target gene–TF network. The nodes in the orange circle are genes, and the nodes in the blue circle are TFs. (c) Drug–gene interaction network. The orange circled node is the gene, and the green circled node is the drug. (d) Target gene–miRNA network of NUFIP2, CCND2, ZNF264, and hsa-mir-1-3p. (e) Target gene–TF network of ACBD4, BAX, CCDC142, AKT1S1, and HES4. (f) Drug–gene interaction network containing CYP3A4, KRAS, and NFE2L2.

Supplementary Fig. 3 Screening and verification of biomarkers. (a–b) Least Absolute Shrinkage and Selection Operator (LASSO) logistic regression algorithm to screen biomarkers. Different colors represent different genes. (c–d) RFB algorithm to screen biomarkers. (e) Venn diagram shows the intersection of the biomarkers obtained by the two algorithms. (f) ROC curve of HDAC9 and ARRDC4 in GSE78721. (g) ROC curve of HDAC9 and ARRDC4 in the validation set GSE76894. (h) ROC curve of HDAC9 and ARRDC4 in the validation set GSE9006.

Supplementary Fig. 4 Correlation analysis of hub genes. (a) The expression levels of hub genes in the three characteristic T2D subtypes, the

blue is the C1 subtype, the orange is the C2 subtype, and the red is the C3 subtype. The horizontal axis is the level of gene expression, the vertical axis is the genes, and the color bars represent the three subtypes. (b–d) Heat map of the correlation between hub genes and 29 immune-related gene sets in three subtypes. The size of the colored square represents the strength of the correlation; the blue indicates negative correlation and red indicates positive correlation. The darker the color, the stronger the correlation.

Supplementary Fig.5 Correlation analysis between diagnostic markers and immune cells infiltration (a) The correlation between HDAC9 and immune cell infiltration. (b) The correlation between ARRDC4 and immune cell infiltration.

Supplementary Table 1. The information summary of GEO dataset.

Supplementary Table 2. Differential gene expression (DEGs) table.

Supplemantary Table 3. GO/KEGG enrichment analysis results.

Supplementary Table 4. GSEA analysis results.

Supplementary Table 5. Results of GSVA analysis.

Supplementary Table 6. target genes - miRNA regulatory network topology Table.

Supplementary Table 7.target genes - TF regulatory network topology Table.

Supplementary Table 8. Fasting blood glucose, insulin and QUICKI of

mice in each group.
